# Peri-implant Sulcular Fluid Galectin-1, Soluble Urokinase Plasminogen Activator Receptor and IL-1β Levels under Peri-implant Inflammatory Conditions

**DOI:** 10.3290/j.ohpd.b2082081

**Published:** 2021-09-30

**Authors:** Ibraheem F. Alshiddi, Abdulrahman M. AlMubarak, Montaser N. Alqutub, Firas K. Alqarawi, Faris A. Alshahrani, Fawad Javed, Fahim Vohra, Tariq Abduljabbar

**Affiliations:** a Associate Professor, Department Of Prosthetic Dental Science, College of Dentistry, King Saud University, Riyadh, Saudi Arabia. Performed the clinical examinations and wrote the manuscript.; b Assistant Professor,Department of Periodontics and Community Dentistry, College of Dentistry, King Saud University, Riyadh, Saudi Arabia. Performed the laboratory-based investigations and wrote the manuscript.; c Associate Professor,Department of Periodontics and Community Dentistry, College of Dentistry, King Saud University, Riyadh, Saudi Arabia. Performed the radiographic examinations, revised the manuscript.; d Assistant Professor, Department of Substitutive Dental Sciences, College of Dentistry, Imam Abdulrahman Bin Faisal University, Dammam, Saudi Arabia. Wrote the introduction and methods.; e Assistant Professor, Department of Substitutive Dental Sciences, College of Dentistry, Imam Abdulrahman Bin Faisal University, Dammam, Saudi Arabia. Administered the questionnaire to the participants, wrote the results.; f Assistant Professor, Department of Orthodontics and Dentofacial Orthopedics, Eastman Institute for Oral Health, University of Rochester, NY, USA. Wrote the manuscript and revised it prior to submission.; g Professor, Department of Prosthetic Dental Science, College of Dentistry, King Saud University, Research Chair for Biological Research in Dental Health, College of Dentistry, Riyadh, Saudi Arabia. Performed the statistical analysis, wrote the manuscript.; h Professor, Department of Prosthetic Dental Science, College of Dentistry, King Saud University, Research Chair for Biological Research in Dental Health, College of Dentistry, Riyadh, Saudi Arabia. Designed and supervised the project, wrote the manuscript.

**Keywords:** interleukin-1β galectin-1, peri-implant diseases, peri-implant mucositis, urokinase plasminogen activator receptor

## Abstract

**Purpose:**

Soluble urokinase plasminogen activator receptor (suPAR) and interleukin 1-beta (IL-1β) are inflammatory biomarkers, whereas galectin-1 is an anti-inflammatory cytokine. The relationship between suPAR, galactin-1 and IL-1β levels in peri-implant sulcular fluid (PISF) in relation to dental implants remains unaddressed. The aim was to assess suPAR, galectin-1, and IL-1β levels in PISF under peri-implant inflammatory conditions.

**Materials and Methods:**

Demographic data and information related to jaw location and duration of implants in function as well as systemic health was retrieved from patients’ dental records. Peri-implant plaque and gingival indices (PI and GI, respectively), probing depth (PD) and crestal bone loss (CBL) were recorded. The PISF was collected and levels of suPAR, galectin-1 and IL-1β were determined using standard techniques. Sample-size estimation and statistical analyses were done. Correlation of suPAR and galectin-1 with IL-1β were assessed via logistic regression. p-values < 0.05 were considered statistically significant.

**Results:**

Seventy-two patients (45 males and 27 females) with peri-implant diseases were included. Thirty-six patients (22 males and 14 females) had peri-implant mucositis; 36 (23 males and 13 females) had healthy peri-implant tissues. The PISF volume was statistically significantly higher among patients with (0.52 ± 0.05 µl) than without peri-implant diseases (0.06 ± 0.01 µl) (p < 0.001). The PISF levels of suPAR (p < 0.01), galectin-1 (p < 0.01) and IL-1β (p < 0.01) were statistically significantly higher among patients with than without peri-implant diseases. In patients with peri-implant mucositis, PISF suPAR (p < 0.001) and galectin-1 (p < 0.001) levels correlated with PISF IL-1β levels. In patients with peri-implant mucositis, increasing peri-implant PD and IL-1β levels directly correlated with increased PISF suPAR (p < 0.001) and galectin-1 (p < 0.05) levels.

**Conclusion:**

Increased PISF levels of suPAR, galectin and IL-1β suggest that these proteins possibly contribute towards the pathogenesis of peri-implant inflammation, and are potential biomarkers of peri-implant diseases.

Under peri-implant inflammatory conditions, the peri-implant sulcular fluid (PISF) expresses destructive inflammatory (e.g. interleukin-1beta [IL-1β] and tumor necrosis factor-alpha [TNF-α]) and anti-inflammatory cytokines (e.g. IL-4, -10 and -13) in elevated concentrations compared with healthy peri-implant tissues.^1,15,22,41^ Such biological mediators play a role in comprehending the aetiopathogenesis and progression of periodontal and peri-implant inflammatory conditions.^2,3,6,15^

The urokinase-type plasminogen activator receptor (also known as CD87) is a multidomain glycoprotein, which tethers to cell membranes of a variety of cells, including activated T-lymphocytes, endothelial cells, fibroblasts, macrophages, monocytes, and certain tumor cells.^18,33^ Release of this protein from the membranes results in the formation of soluble urokinase plasminogen activator receptor (suPAR).^33,43^ Depending upon the severity of inflammation, suPAR is expressed in various body fluids, such as plasma, urine and cerebrospinal fluid.^38,42^ SuPAR has also been identified in unstimulated whole saliva and gingival crevicular fluid samples collected from periodontally healthy adults.^24,42^ In this regard, suPAR has been suggested as a biomarker of inflammation.^40^ Galectin-1 is an anti-inflammatory cytokine released from various cells, including dendritic cells, fibroblasts, macrophages, endothelial cells, and B- and T-lymphocytes.^10,36,39^ Galectin-1 participates in various biological events such as immunomodulation, metastasis, and cellular adhesion and growth regulation.^37,44^ Results from an experimental study in mice showed that galectin-1 facilitates resolution of inflammation and may help control uncontrolled inflammatory disorders. Furthermore, increased GCF (gingival crevicular fluid) IL-1β levels have been associated with the aetiopathogenesis and progression of cardiovascular and periodontal diseases.^20,32,35^ In a recent study, Taşdemir et al^42^ assessed the GCF suPAR and galectin-1 levels among patients with gingivitis and periodontitis and compared them with periodontally healthy individuals (controls). The results showed that GCF supAR and galectin levels were significantly higher among patients with gingivitis and periodontitis compared with controls;^42^ however, there was no difference in the GCF levels of these proteins when compared between patients with gingivitis and periodontitis.^42^ The study concluded that suPAR and galectin-1 are potential biomarkers of periodontal disease and may play a role in the development and prognosis of periodontal disease.

Periodontal and peri-implant diseases share common risk factors (such as poor oral hygiene, tobacco smoking and immunosuppression)^12,23,28^ as well as common microbiological, histologic and immunobiological pathways of disease progression.^9,13,26,31^ The present authors hypothesise that PISF suPAR and galectin-1 levels are higher among patients with peri-implant inflammation, and that levels of these proteins are correlated with the peri-implant probing depth (PD) and PISF IL-1β levels. The aim was to assess PISF suPAR, galectin-1, and IL-1β levels under peri-implant inflammatory conditions.

## Materials and Methods

### Ethics Declaration

Guidelines documented in the Helsinki-2013 Declaration of experiments on humans were adopted for this study. Participating individuals were required to read and signed a consent form. Prior to signing the consent form, all participating patients were informed that they could withdraw from the study at any stage without any penalty, and were invited to ask questions. The ethics committee of Centre for Specialist Dental Practice and Clinical Research (UDCRC/ 029/20) approved the study protocol. Signing the consent form was mandatory for all participants.

### Participants and Eligibility Criteria

The study was conducted between July 2020 and January 2021. All patients were recruited from the Specialist Care Centre in Riyadh, Saudi Arabia. Patients having undergone dental implant therapy for partial and/or complete edentulism were invited. Individuals habitually using alcohol and nicotinic products such as waterpipe, cigarettes, pipe, cigarillos and electronic nicotine delivery systems (ENDS), patients with self-reported systemic diseases such as diabetes mellitus (DM), cardiomyopathy, obesity, HIV/AIDS, or renal diseases and hepatic disorders were excluded. Moreover, implants placed in grafted sites were not assessed. Furthermore, pregnant/lactating individuals and individuals using bisphosphonates, probiotics, antibiotics, steroids, and non-steroidal anti-inflammatory drugs were excluded.

### Study Groups

Based upon the peri-implant clinicoradiographic status, participants were divided into 2 groups: (a) patients with peri-implant diseases and (b) patients without peri-implant diseases. With reference to Consensus Report of Workgroup 4 of the 2017 World Workshop on Classification of Periodontal and Peri-implant Diseases and Conditions, the following parameters were used to diagnose peri-implant diseases: (a) marginal bone loss (MBL) of at least 3 mm; (b) presence of suppuration or bleeding and/or SUP; (c) PD ≥6 mm.^16^ Using the criteria mentioned above,^16^ peri-implant diseases were categorised as peri-implant mucositis or peri-implantitis.

### Evaluation of Patient Records

Information pertaining to patients’ age, gender, and daily brushing and flossing habits was collected. Data pertaining to duration of implants in function, implant geometry (platform switching, length and diameter), jaw location, implant loading protocol (immediate, early or delayed loading), insertion torque used, mode of prosthesis retention (screw or cement retention) and depth of insertion (crestal or subcrestal) was retrieved from patients’ records. This information was collected by one trained investigator.

### Peri-implant Clinicoradiographic Parameters

Peri-implant mPI,^34^ mGI,^34^ and PD^27^ were measured at 6 sites/implant (distobuccal, distopalatal/distolingual, midbuccal, mesiobuccal, midlingual/midpalatal and mesiolingual/mesiopalatal) by a trained and calibrated author (FV; Kappa score 0.92). A graded plastic probe (Hu-Friedy; Chicago, IL, USA) was used to determine the periimplant PD. Digital bitewing radiographs (Soredex; Helsinki, Finland) were taken at 66 kVp and 8 mA for digital assessment of crestal bone loss (CBL). The CBL was defined as the vertical distance from 2 mm below the implant-abutment interface to the crest of jaw bone on the mesial and distal surfaces of adjacent implants.^28^ The CBL was measured on the distal and mesial surface of each implant by one trained and calibrated investigator (Kappa score 0.94).

### Collection of PISF

The PISF samples were collected approximately 48 h after the clinicoradiographic evaluation, with the patients in a fasting state. All PISF samples were collected during the morning (between 8:00 and 9:00 am) by a trained and calibrated investigator (Kappa 0.88). The PISF collection was performed according to the protocols described in the studies by Alqahtani et al^11^ and Bhardwaj et al.^17^ Briefly, patients were comfortably seated on a dental unit and a saliva ejector was placed in the mouth. Sterile cotton tips were used to gently remove the supragingival plaque from the implant surface. The site was gently dried with a triple syringe and isolated with sterile cotton-rolls. Sterile standard paper strips (Periopaper, Interstate Drug Exchange; Amityville, NY, USA) were placed in the deepest buccal pocket of the peri-implant sulcus for 0.5 min. In patients with two or more implants, PISF was collected from the implant that displayed the deepest buccal PD. Saliva- and/or blood-contaminated paper strips were discarded, and the sampling protocol was repeated after 60 min. The volume of the collected PISF was determined using a calibrated device (Periotron 8000, Oraflow; Amityville, NY, USA). Samples were stored at −80°C before biochemical analysis.

### Assessment of suPAR, galectin-1, and IL-1**β**

Levels of suPAR (Human suPAR ELISA kit, MBS7606253, MyBiosource; San Diego, CA, USA), galectin-1 (RayBiotech, Human GAL-1, ELH-galectin-1; Norcross, GA, USA) and IL-1β (SunRed Bio; Shanghai, China) in the PISF were determined using enzyme-linked immunosorbent assay according to the manufacturer’s guidelines. Solutions of standard and samples were added into the wells precoated with IL-1β and suPAR antibodies. To each well, streptavidin-conjugated HRP enzyme and biotin conjugated antibody were added. The plates were incubated for 60 min at 37°C, following which the wells were washed 5x with 350 μl of a wash buffer. Chromogen was added and the plates were again incubated at 37°C in a dark room. The reaction was completed using a H_2_SO_4_ stop solution. To assess the galectin-1 levels in the PISF, standards and samples were added to the wells and the plates were incubated for 1.5 h at 37°C. Biotin conjugated antibody was added to the wells and the plates were incubated for 60 min at 37°C. The plates were washed 3x with 350 μl of a wash buffer, and streptavidin HRP was added to the wells. After an incubation period of 30 min at 37°C, the plates were washed 5x with 350 µl of a wash buffer. The substrate for the HRP enzyme was added and plates were incubated in a dark room at 37°C. The reaction was completed using H_2_SO_4_ as stop solution. An ELISA reader (BioTek, ELX 800) was used at 450 nm to read the absorbances of all biomarkers, and concentrations of samples were calculated based on standard absorbance values.

### Sample-size Estimation and Statistical Analyses

Power analysis was done using a computer program (G*Power version 3.1.5., University of Kiel, Kiel, Germany) and was based on the results of a pilot investigation and results of a previous study^42^ with similar objectives. A t-test of independent means (test and control groups) was set as the statistical test to perform power analysis using an effect size and alpha of 1 and 5%, respectively. Correlation of suPAR and galectin-1 with IL-1β were assessed via logistic regression. It was estimated that inclusion of at least 32 individuals per group was necessary to attain a study power of 90%. Logistic regression analysis was performed to assess the correlation of suPAR and galectin-1 levels with PD and PISF IL-1β levels. A software program (IBM SPSS v 20; Chicago, IL, USA) was used to compare clinicoradiographic and biological parameters among patients with and without peri-implant diseases. Groups were compared using Student’s t-test and the Mann-Whitney U-test. A p-value less than 0.05 was set as an indicator of statistical significance.

## Results

### Demographics

Thirty-six patients (22 males and 14 females) with and 36 (23 males and 13 females) without peri-implant diseases were included. The mean ages of individuals with and without peri-implant diseases were 60.6 ± 5.7 and 59.3 ± 3.5 years, respectively. There was no statistically significant difference in the mean age of males and females in either group (Table 1). Toothbrushing twice daily was more often reported by individuals without (n = 31) than with (n = 8) peri-implant diseases. Daily flossing of interproximal spaces was reported by 25 individuals without peri-implant diseases and none of the patients with peri-implant diseases

**Table 1:** Characteristics of the study groups

Parameters	Patients with peri-implant disease	Patients without peri-implant diseases
Number of patients	36	36
Males:Females	22:14	23:13
Mean age (all patients)	60.6 ± 5.7 years	59.3 ± 3.5 years
Mean age (males)	63.4 ± 5.1 years	62.2 ± 4.7 years
Mean age (females)	57.1 ± 2.8 years	56.4 ± 2.7 years
Toothbrushing		
Once daily	28	5
Twice daily	8	31
Daily flossing	None	25

### Dental Implants

In total, 36 (16 in maxilla and 20 in the mandible) and 36 (18 in maxilla and 18 in the mandible) dental implants were in place in patients with and without peri-implant diseases, respectively. In patients with peri-implant diseases, 20 and 16 implants were located posterior maxilla and mandible, respectively. In patients without peri-implant diseases, 16 and 18 implants were located in the posterior maxilla and mandible, respectively, and 2 implants were present in the anterior maxilla. All implants were cement-retained, delayed-loaded and placed at bone level using insertion torques ranging between 30 and 35 Ncm. All implants were platform-switched with diameters and lengths ranging between 4-4.1 and 11-14 mm, respectively (Table 2). Scores of peri-implant mPI (modified plaque index, p < 0.01), mGI (modified gingival index; p < 0.01) and PD (p < 0.01) were significantly higher among patients with than without peri-implant diseases (Table 3). All patients with peri-implant diseases were diagnosed with peri-implant mucositis.

**Table 2:** Characteristics of implant placed in the study groups

Implant parameters	Patients with peri-implant disease	Patients without peri-implant diseases
Number of implants (n)	36	36
Maxilla:mandible (n)	16:20	18:18
Implant distribution		
Anterior maxilla	0	2
Posterior maxilla	20	16
Anterior mandible	0	0
Posterior mandible	16	18
Insertion torque	30–35 Ncm	30–35 Ncm
Implant dimensions (D x L)	4–4.1 mm x 11–14 mm	4–4.1 mm x 11–14 mm
Duration in function in years	3.3 ± 0.4	3.1 ± 0.2
Loading protocol	Delayed loading	Delayed loading
Depth of insertion	Bone-level	Bone-level
Placement at grafted sites	None	None
Implant geometry	Tapered and platform switched	Tapered and platform switched

**Table 3:** Characteristics of implant placed in the study groups

Implant parameters	Patients with peri-implant disease	Patients without peri-implant diseases
Modified plaque index	2.04 ± 0.06*	0.4 ± 0.003
Modified gingival index	2.23 ± 0.2*	0.2 ± 0.006
Probing depth (in mm)	4.7 ± 0.2*	1.6 ± 0.04
Crestal bone loss (mesial surface)	0.6 ± 0.07 mm	0.3 ± 0.006 mm
Crestal bone loss (distal surface)	0.5 ± 0.04 mm	0.2 ± 0.002 mm

*Compared with patients without peri-implant diseases (p < 0.01).

### PISF Volume and suPAR, Galectin-1 and IL-1**β** Levels

The volume of collected PISF was significantly higher among patients with (0.52 ± 0.05 µl) than without peri-implant diseases (0.06 ± 0.01 µl) (p < 0.001). Table 4 shows the levels of suPAR, galectin-1 and IL-β in the PISF of patients with and without peri-implant diseases. The PISF suPAR (p < 0.01), galectin-1 (p < 0.01) and IL-1β (p < 0.01) levels were significantly higher among patients with peri-implant diseases than among individuals with a healthy peri-implant clinicoradiographic status. There was no statistically significant influence of gender and jaw location on the levels of suPAR, galectin-1 and IL-β in the PISF of patients with and without peri-implant diseases.

**Table 4:** Levels of suPAR, galectin-1 and IL-β in the PISF

Parameters	suPAR	Galectin-1	IL-1β
Patients with peri-implant diseases	4.32 ± 0.24 ng/ml*	38.6 ± 4.3 ng/ml*	406.3 ± 23.4 ng/ml*
Males	4.51 ± 0.17 ng/ml*	45.3 ± 8.1 ng/ml*	387.4 ± 20.7 ng/ml*
Females	4.08 ± 0.35 ng/ml*	36.7 ± 5.2 ng/ml*	420.5 ± 25.7 ng/ml*
Maxilla	4.61 ± 0.15 ng/ml*	40.5 ± 2.6 ng/ml*	366.9 ± 30.4 ng/ml*
Mandible	4.24 ± 0.22 ng/ml*	33.6 ± 1.7 ng/ml*	431.2 ± 24.1 ng/ml*
Patients without peri-implant diseases	0.25 ± 0.08 ng/ml*	5.2 ± 0.6 ng/ml*	58.6 ± 10.2
Males	0.22 ± 0.08 ng/ml*	3.6 ± 0.7 ng/ml*	50.4 ± 9.6 ng/ml*
Females	0.28 ± 0.04 ng/ml*	5.7 ± 0.3 ng/ml*	61.3 ± 3.1 ng/ml*
Maxilla	0.28 ± 0.05 ng/ml*	4.2 ± 0.2 ng/ml*	55.1 ± 3.3 ng/ml*
Mandible	0.24 ± 0.06 ng/ml*	5.5 ± 0.6 ng/ml*	62.4 ± 5.2 ng/ml*

*Compared with patients without peri-implant diseases (p < 0.01).

### PISF suPAR and Galectin-1 Levels in Correlation with IL-1**β** and PD

**Fig 1:**
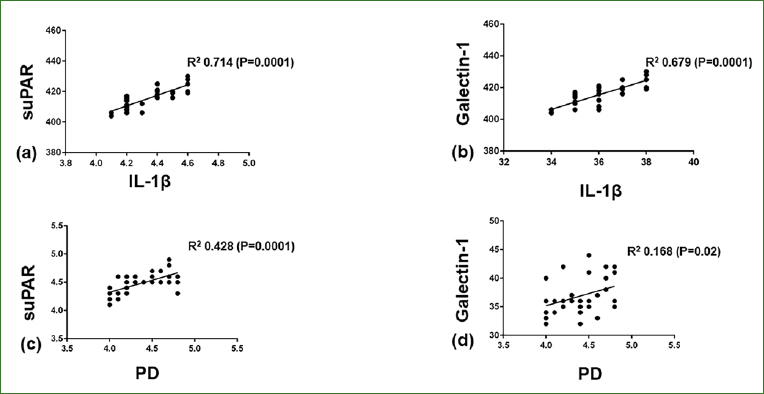
Correlation of suPAR and Galectin-1 with IL-1b levels in the peri-implant sulcular fluid and probing depth in patients with peri-implant mucositis.

**Fig 2:**
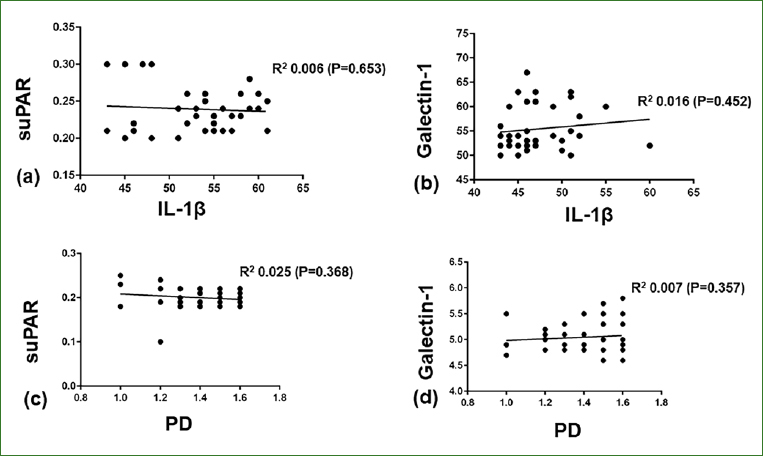
Correlation of suPAR and Galectin-1 with IL-1b levels in the peri-implant sulcular fluid and probing depth in patients without peri-implant mucositis.

In patients with peri-implant mucositis, PISF suPAR (p < 0.001) and galectin-1 (p < 0.001) levels correlated with PISF IL-1β levels. In patients with peri-implant mucositis, increasing peri-implant PD and IL-1β levels directly correlated with increased PISF suPAR (p < 0.001) and galectin-1 (p < 0.05) levels (Fig 1). In patients without peri-implant diseases, there was no significant correlation of PISF suPAR and galectin-1 levels with PD and PISF IL-1β levels (Fig 2). In patients without peri-implant diseases, there was no statistically significant relationship between peri-implant PD and IL-1β levels and suPAR and galectin levels (data not shown).

## Discussion

The present study hypothesis was that PISF suPAR and galectin-1 levels are higher among patients with peri-implant inflammation and that levels of these proteins are correlated with the peri-implant PD and PISF IL-1β levels. To the authors’ knowledge, this is the first power-adjusted study in which levels of suPAR and galectin-1 were assessed in the PISF. Our results are in accordance with the proposed hypothesis, as the PISF suPAR and galectin levels were nearly 18x and 7x higher, respectively, among patients with peri-implant inflammation compared with controls. Moreover, increasing peri-implant PD and PISF IL-1β levels significantly correlated with an increased expression of suPAR and galectin-1 among patients with peri-implant inflammation. The present regression analysis results reflect that an increased PD is correlated with higher PISF IL-1β levels or vice versa in patients with peri-implant mucositis. However, this finding does not agree with previous studies^5,30^ that have reported otherwise. In the study by Kajale and Mehta,^30^ no statistically significant association was identified between PISF IL-1β levels and clinicoradiographic parameters, whereas according to Al-Ali et al,^5^ PISF IL-1β levels statistically significantly corelated with peri-implant CBL. It is important to interpret these results cautiously, as the sample in the study by Kajale and Mehta^30^ was not power-adjusted and the participants in the study by Al-Ali et al^5^ had peri-implantitis. Based upon the clinical and immunoinflammatory outcomes of the present study, it is anticipated that in patients with peri-implant mucositis, increasing PD correlates with the expression of IL-1β and suPAR in the PISF, which in turn increases the PISF galectin-1 levels to repulse the peri-implant inflammatory burden. Further studies are needed to investigate the reproducibility of the present results.

In the present study, none of the patients with peri-implant diseases had peri-implantitis. The authors of the present power-adjusted study would like to emphasise that by no means was the presence of peri-implantitis an exclusion criterion. One logical justification for the absence of peri-implantitis in the target population assessed could be linked with the relatively short duration of implants in function (approximately 3 years) in both groups. According to Albrektsson et al,^8^ CBL of up to 2 mm after the first year of loading followed by a yearly CBL of 0.2 mm is considered normal. In the present study, the average CBL among implants in both groups was approximately 2 mm, which is in accordance with the study by Albrektsson et al.^8^ It is hypothesised that dental implants that have been in function for at least 10 years demonstrate significantly more CBL compared with dental implants that have been in function for relatively short durations (up to 3 years). The authors of the present study speculate that levels of suPAR, galactin-1 and IL-1 β in the PISF are higher among patients with peri-implantitis than among individuals with peri-implant mucositis and those with a healthy peri-implant clinicoradiographic status. However, in a recent clinical study, Taşdemir et al^42^ evaluated the levels of suPAR and galectin-1 in the GCF of patients with gingivitis, and periodontitis and compared them with individuals with a healthy periodontal status. The results showed that concentrations of suPAR and galectin-1 were significantly higher in the GCF of patients with periodontitis and gingivitis compared with periodontally healthy controls. Moreover, the authors^42^ reported no significant difference in the GCF levels of suPAR and galectin-1 among patients with periodontitis and gingivitis. Based upon the results reported by Taşdemir et al,^42^ it remains to be determined whether or not PISF suPAR and galectin-1 levels differ among patients with peri-implant mucositis and peri-implantitis. Further studies are needed in this regard.

## Conclusion

Increased PISF levels of suPAR, galectin and IL-1β suggest that these molecules play a role in the pathogenesis of peri-implant inflammation, and are potential biomarkers of peri-implant diseases.
